# A learning based approach for designing extended unit cell metagratings

**DOI:** 10.1515/nanoph-2021-0540

**Published:** 2021-12-08

**Authors:** Soumyashree S. Panda, Ravi S. Hegde

**Affiliations:** Department of Electrical Engineering, IIT Gandhinagar, Gandhinagar, 382355, India

**Keywords:** color filters and splitter, deep learning, evolutionary optimization, inverse design, metagratings, metasurface design

## Abstract

The possibility of arbitrary spatial control of incident wavefronts with the subwavelength resolution has driven research into dielectric optical metasurfaces in the last decade. The unit-cell based metasurface design approach that relies on a library of single element responses is known to result in reduced efficiency attributed to the inadequate accounting of the coupling effects between meta-atoms. Metasurfaces with extended unit-cells containing multiple resonators can improve design outcomes but their design requires extensive numerical computing and optimizations. We report a deep learning based design methodology for the inverse design of extended unit-cell metagratings. In contrast to previous reports, our approach learns the metagrating spectral response across its reflected and transmitted orders. Through systematic exploration, we discover network architectures and training dataset sampling strategies that allow such learning without requiring extensive ground-truth generation. The one-time investment of model creation can then be used to significantly accelerate numerical optimization of multiple functionalities as demonstrated by considering the inverse design of various spectral and polarization dependent splitters and filters. The proposed methodology is not limited to these proof-of-concept demonstrations and can be broadly applied to meta-atom-based nanophotonic system design and in realising the next generation of metasurface functionalities with improved performance.

## 1 Introduction

State-of-the-art nanofabrication technologies with extraordinary lateral resolution and high stitching accuracy now enable the realization of wide area precision nanostructures. In combination with advanced deposition technologies which provide great control over film thickness and uniformity, close and precise stacking of nanostructured films is also possible. A wide range of material choices, e.g., plasmonic materials (Au, Ag etc.), high index dielectrics (Si, Ge, GaAs, SiN, TiO_2_ etc.), graphene and other 2D materials, phase change materials and transparent conducting oxides complement the large number of spatial degrees of freedom available in nanopatterning. The optical metasurface, a spatially heterogeneous array of nanoresonators is one example of the devices that are now within the reach of nanofabrication with the tantalizing possibility of full local control of an incident wavefront’s properties (amplitude, phase, spectrum or polarization state). Such exquisite control in conjunction with active tunability can enable a new class of ultra-thin, actively controllable, optical devices with a variety of functionalities like beam shaping [1], lensing [2–4], beam steering [5–7], polarizing [8, 9] and positively impact diverse applications like imaging [10], sensing [11], holography [12, 13] and optical computing [14].

However, for designing a broad class of nanophotonic systems, including photonic crystals, metasurfaces, and plasmonic structures, the motif of a unit cell with a single resonator is predominantly employed owing to its simplicity. For the optical metasurface, the so-called unit-cell approximation creates a library that maps the geometrical parameters of a resonator to the optical response of a periodic metasurface consisting of this meta-atom. The final design is then arrived at by simply stitching together various library elements. The limitations of this design approach have been recently explored in the nanophotonics literature [15–18]. Gigli et al. [19] demonstrated the infeasibility of quasi-independent operation with weak crosstalk for nanoresonators arrayed with a sub-wavelength or even wavelength-scale distance. The limitations are most noteworthy in the case of beam shaping and lensing applications. For instance, the numerical calculations by Gigli et al. show that the efficiency of the Huygens’ elements remains below 40% for monochromatic operation, even for meta-gratings with periods significantly larger than the wavelength. This performance reduction is now well known in the field and is ascribed to inter-element electromagnetic coupling [20–22].

To overcome these limitations, local periodization [22, 23] and global optimization techniques [21, 24] have been explored and found to give performance improvements. Specifically, metagratings capable of higher beam bending angles in transmission [25–27] and reflection [28, 29] mode have been proposed. The idea behind these metagratings is to break the symmetry of illumination conditions and channelize light into diffraction orders by engineering their scattering patterns. Such extended unit-cell geometries, where high-index dielectric nanoantennas feature multiple geometry-tunable resonances [30–33], have enabled selective enhancement/suppression of single diffraction order in both reflection and transmission modes. A second aspect is that the extension of the unit cell with multiple meta-atoms drastically enlarges the parameter space and consequently provides potential configurations with improved device performance. While extended-unit cell designs using the concept of meta-atom multiplication do not induce additional complexity for lithography-based fabrications, the substantially increased number of parameters makes the design methodology based on physical intuition and parameter sweep impractical. The presence of multiple diffraction orders in metagratings and the absence of easily discernible empirical relationships between geometries and optical properties make it an especially challenging problem. Multiple separate inclusions have an inherent frequency divergence that hinders the high diffraction efficiency from wide frequency and angle ranges, which are highly desirable for beam steering applications. Asymmetric nanoantenna separation is also strongly correlated to diffraction efficiency, which raises the challenge of stringent fabrication precision.

In this article, we propose an improved learning based nanophotonic structure discovery methodology for the constrained inverse design of extended unit-cell metagratings. Inverse design with adequate accounting of fabrication constraints is an optimization occurring in a constrained higher dimensional parameter space. The computational cost of such optimization scales exponentially with linear scaling in the parameter space dimensionality. This is often termed as the “curse of dimensionality” in the optimization literature [34]. This has opened a new computation-intensive front for nanophotonic research [35–38]. Machine learning, in particular, deep learning [39, 40] (DL), is being increasingly investigated for inverse design problems in photonics [41–45]. DL based inverse design [46] has been explored for a broader class of photonics problems [41, 47]: nanoresonators [48, 49], plasmonic structures [50–52], metasurfaces/metamaterials [50–66], integrated photonics [67, 68] and topological photonics [50], [51], [52, 61], [62], [63], [64], [65], [66, 69]. As DL begins to be increasingly applied in the inverse design of complex structures with high forward simulation load, feasible datasets may be “small” with potentially significant biasing due to the dataset construction. Thus, there is a need for workflows that not only emphasize efficient sample generation but also reduce the burden involved in learned model creation.

We consider the complex multi-variable geometry of rectangular unit-cells with multiple non-intersecting elliptical nanopillars [33] for wide-band multi-order functionalities. Previous reports [70–73] have limited the structure discovery to a narrow-band wavelength-limited space (as reported by Inampudi and coworkers [55, 72, 74, 75]) or diffraction order limited space (as reported by Kiarashinejad and coworkers [73, 76, 77]). In creating good models, we found that a systematic study of network architectures and techniques to limit the amount of ground-truth training data was essential. The learning methodology typically employed in the literature has overwhelmingly favored simple fully-connected feedforward neural network architectures, randomly sampled training datasets and model performance estimation using testing sets sampled identically to the training dataset. The commonly used feedforward architectures suffer from the well-known issue of vanishing gradients which leads to a saturated prediction performance with increasing network depth. Residual layers implemented by Jiang and coworkers [78] and Yeung and coworkers [79] are adopted here for addressing this shortcoming along with Dense network architectures (DenseNet). Greedy sampling and centroid based sampling strategies are compared to random dataset sampling. The prediction performances of trained models on unseen data are investigated by building surrogate models and implementing them in surrogate-based evolutionary optimization.

The paper is organized as follows. Following this introduction, a detailed discussion of problem description and design approach is presented in Section 2 including problem encoding (subsection 2.2.1), ground truth generation (subsection 2.2.2) and learned model creation (subsection 2.2.3). Methods to estimate the global prediction capability of models via surrogate-assisted optimization are discussed in subsection 2.2.4. The results and discussion section (Section 3) begins with global prediction abilities of the surrogate models. Surrogate assisted optimizations are discussed in subsection 3.1 to design different spectral filters before concluding in Section 4.

## 2 Problem description and design approach

### 2.1 The extended unit-cell metagrating

**Figure 1:**
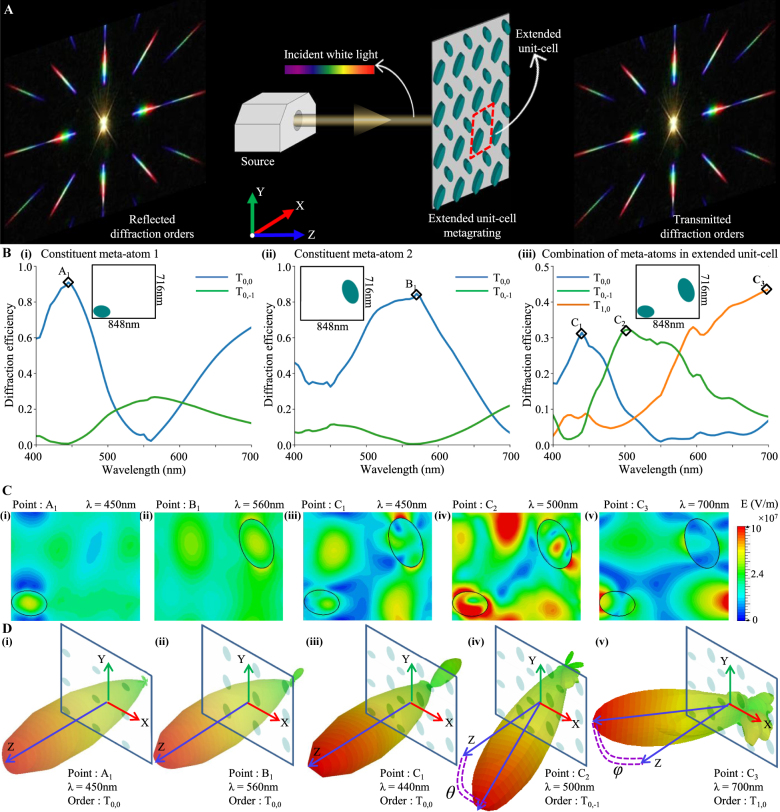
The extended unit cell metagrating and its optical response. (A) Schematic of the extended unit-cell metagrating and scattering patterns of a metagrating dividing lights equally among orders when subjected to white light. Both transmitted and reflected diffraction orders are shown. (B) Comparison of diffraction efficiencies of three metagratings across different diffraction orders. The metagratings are constructed by periodically extending (i) first constituent meta-atom in a unit-cell, (ii) second constituent meta-atom in a unit-cell and (iii) both meta-atoms in an extended unit-cell. Peak diffraction efficiencies are marked. (C) E field distributions and (D) scattering power patterns corresponding to marked points in B are shown.

The building block of the metagratings proposed in this work is an extended unit cell that is composed of two nonidentical non-intersecting elliptical TiO_2_ nanopillars. The configuration results in the coexistence of multipoles of high orders when arranged in a periodic fashion. A typical symmetric 2D grating, when subjected to normally incident polarized white light, splits the beams into different diffraction orders uniformly at angles depending on the wavelength and lattice constants (Figure 1A). However, engineering the multipolar interference in an extended unit-cell allows us to control the power distribution in different orders and wavelengths. Building on the seminal works of Shibanuma and coworkers [80], tunable control of directional scattering has been proposed with the help of cylindrical dimers [30], touching dimers [31], elliptical dimers [33] and prismoid-circular dimers [33]. The extended unit cell adapted in this work takes inspiration from the metasurface geometry proposed by Khaidarov and coworkers [33]. In their work, Khaidarov and coworkers considered a unit-cell that consists of asymmetric elliptical dimers in a single unit cell. They have demonstrated that such asymmetric arrangement of elliptical nano-antenna can control the power distribution into various diffraction orders. Such arrangements can help achieving a variety of wavelength and polarization-dependent functionalities like filtering [32], beam deflection [81], beam splitting etc.

The optical behavior of a typical extended unit-cell metagrating is shown in Figure 1B-iii. The spectral behavior of two other metagratings designed by periodically extending only one of the constituent meta-atom is shown in Figure 1B-i and ii. Both constituent meta-atoms are observed to be giving a peak diffraction efficiency in (0,0)^th^ transmission order at wavelength 450 and 560 nm, respectively, with negligible efficiency in other orders; whereas, the extended unit-cell gives diffraction peaks in the (0,0)th, (0,−1)th and (1,0)th orders at 450, 500 and 700 nm, respectively. The E-field distributions for these marked diffraction peaks are shown in Figure C-i–Figure 1C-i–v. For single-atom unit cells (Figure 1C-i and ii), fundamental electric dipole modes can be observed inside the resonators which can be determined with the help of Mie theory in the limit of large index contrast [82]. However, for extended unit-cells (Figure C-iii–Figure 1C-iii–v), hybrid modes are observed which do not show an easily discernible relationship with individual particle resonators. This coexistence of multipoles leads to the asymmetrical farfield scattering patterns shown in Figure D-i–Figure 1D-i–v.

### 2.2 Design approach

As noted in subsection 2.1, the behavior of an extended unit-cell meta-atom cannot be easily expressed in terms of the optical behavior of constituting resonators when considered in isolation. The proposed inverse design process is carried out by an optimization algorithm that searches the full geometrical parameter space of the extended unit-cell with the help of a learning based prediction model. Using a prediction model in place of an EM solver significantly accelerates the numerical optimization with reduced computational cost, but requires a one-time computational investment for ground-truth dataset generation and subsequent DNN model training. Due to the high dimensionality of the problem, special care is given in smart-sampling the parameter space and design of efficient network architectures. The DL enabled surrogate optimization process can be divided into three steps: (1) choosing a suitable problem encoding; (2) ground truth generation; (3) choosing a model architecture and learned model creation; and (4) inverse design using the learned model. In this section, each of these steps is described briefly with further details provided in the supporting document.

#### 2.2.1 Problem encoding

The geometry considered here (Figure 1A) is an extended unit cell [32] metagrating consisting of two elliptical TiO_2_ nano-pillars on an SiO_2_ substrate. Each elliptical pillar can be represented by the following set of geometrical parameters: coordinates of the center (*x* and *y* coordinates), major and minor radii (*r*_major_ and *r*_minor_, respectively) and angle of inclination (*θ*) (as shown in the inset of Figure 1A). The geometrical parameters of the two elliptical nanopillars; the lattice periodicities along the *x* and *y* directions (*P*_*x*_ and *P*_*y*_, respectively); and, the metasurface thickness (*t*) form a 13-dimensional parameterized vector. Fixing the *x* and *y* position of the center of one of the ellipses to (0,0) allows us to reduce the dimensionality of the solution space by 2.

The individual parameters in the parameter vector are constrained to lie between their minimum and maximum values and the solution space is a region in the higher dimensional parameter space defined by these constraints. However, this solution space may also contain some structures that are infeasible for nanofabrication. Additionally, some parameter combinations may result in the intersection of the ellipses. Although the structures with intersecting ellipses may be feasible for fabrication, their optical response may greatly differ from the response of structures without such intersections. Therefore, subregions of the solution space are excluded from consideration which contains designs that are infeasible for nanofabrication (which violate lithographic constraints [83] like minimum feature size) and those that lead to intersecting ellipses. Specifically, the minimum value of major and minor radii was set to 50 nm and, to avoid redundancy, *r*_major_ is always taken greater than *r*_minor_. To maintain adequate spacing between ellipses, the ellipses a first scaled to buffer ellipses (indicated by dotted blue lines in the inset of figure: schematic A) such that *r*_buffer_ = *r*_ellipse_ + 10 nm for both major and minor radii. Geometries with intersecting buffer ellipses are left out; which not only ensures a minimum gap of 20 nm between ellipses but also eliminates structures with intersecting ellipses. The buffer ellipses should also not intersect the lattice rectangle; thus providing adequate distance (20 nm) between neighboring unit cells.

The observations in this work are limited to visible wavelength (400–700 nm) and the periodicities of structures are set between 600 and 1000 nm. Only for the periodicities higher than 800 nm we get diffraction into the second order and that too for a shorter range of wavelengths (e.g., 400–500 nm for the largest periodicity of 1000 nm). For all other periodicities and wavelengths, diffraction is only up to the first order. Thus, we limit the observation up to the first diffraction order only. Therefore, we consider 18 diffraction orders (9 in transmission and 9 in reflection) for a single polarization at a given wavelength. Considering two linear polarizations (*s* and *p*) and discretizing the visible spectrum (*λ* = 400–700 nm) with 32 sample wavelengths with 10 nm spacing, we get 36 spectral responses at each of the 32 discrete wavelengths. These numbers constitute the “output” of any learned model. There are various choices for representing this “output”; the approach in this paper is to represent it as a 3D tensor of shape (18,2,32) (see Figure 1B). This choice was motivated by the observation that although the spectral responses of any particular order are smooth functions of wavelength, the splitting various orders at any given wavelength was not very smooth. Cascading them to a 1D tensor causes discontinuities at the points of succession; making the training performance suffer. A 3D tensor representation (Figure 1B) of dimensionality (18,2,32) as chosen in this work not only avoids those discontinuities but also helps to exploit the correlations among output parameters. Additionally, this encoding allows us to use a deconvolution layer toward the end of the network (as shown in Figure 1B) which helps in reducing the number of network trainable parameters. A detailed comparison between the two encoding choices is provided in Section S-1.1 of the supporting document.

#### 2.2.2 Ground-truth generation

As discussed in 2.2.1, the design space is a continuous hypercube in the 11-dimensional continuous solution space (**H**). The imposed constraints reduce the solution space to a “feasible” continuous solution space (**F**) with irregular boundaries (Figure S-2C-i). First we generate a discrete version of the feasible solution space **F**_**s**_ (Figure S-2C-ii) by adequately sampling **F** as follows: *x*, *y* − 100, 200, …, 700, *r*_major_, *r*_minor_ − 50, 100, …, 450, *θ* − 0°, 45°, …, 135°, *P*_*x*_, *P*_*y*_ − 600, 800, 1000 and *t* − 500, 600, 700, 800 with the lengths measured in nanometers. Once generated, samples are selected from **F**_**s**_ and labeled for training the networks. Training and testing datasets include both the input (**X**) and the associated outputs (**Y**) (outputs are termed as “labels”). The labeling process (ground truth generation) is a computationally expensive process. While building a prediction model, a smart selection of datasets (i.e., choosing the **X** array) could lead to a reduction in this burden or lead to a better model for the same computational burden. Three criteria are needed to be considered when selecting samples (**X**) from the design space; *informativeness*, *representativeness* and *diversity* [84]. The conventional ways of sampling are the random sampling [85] (uniform sampling probabilities assigned to all the regions of the solution space) and Latin Hypercube (LHC) sampling [86] (stratifies the input probability distributions). However, due to the irregularities in the distribution of the infeasible designs, LHC sampling does not lead to a representative sampling in our case. In this work, we explore three smart sampling strategies: (1) greedy sampling, (2) k-means clustering and (3) k-medoids clustering to generate the train/test datasets and compare them with random sampling. See Section S-1.2 of the supporting document for further details.

#### 2.2.3 Learned model creation

Three network architectures have been explored in this work and are compared with the most prevalent feedforward architecture; (1) ResNet architecture which is built by cascading multiple residual blocks (a residual block uses a single skip connection in a deep neural network); (2) DenseNet-I architecture where the network is built by cascading dense blocks (a single dense block consists of two skip layers); and (3) DenseNet-II architecture where skip layers are repeated throughout to connect the hidden layers. The architectures are shown in Figure S-2B of the supporting document. In each of these network architectures, the number of hidden layers is increased up to 24 to find the best network configuration. The results are documented in Section S-1.3 of the supporting file. Based on the result given in Figure S-1D, the number of layers is fixed to 13 for feedforward network architectures and 24 for other architectures for the rest of the article. Unless otherwise mentioned, the dense layers consist of 288 neurons.

An extensive comparison of performance was carried out for all combinations of network architectures and sampling strategies discussed above and are documented in Section S-2 of the supporting document. A DenseNet-II network architecture trained with samples selected with the help of k-means clustering strategy is observed to be having a greater prediction ability as compared to other combinations. This prediction model is referred to as “Surrogate-1” in the further part of this work. In order to showcase its efficacy, this model is compared with another prediction model “Surrogate-2” which uses a feed-forward network architecture trained with randomly selected samples.

#### 2.2.4 Surrogate-assisted evolutionary optimization

**Figure 2:**
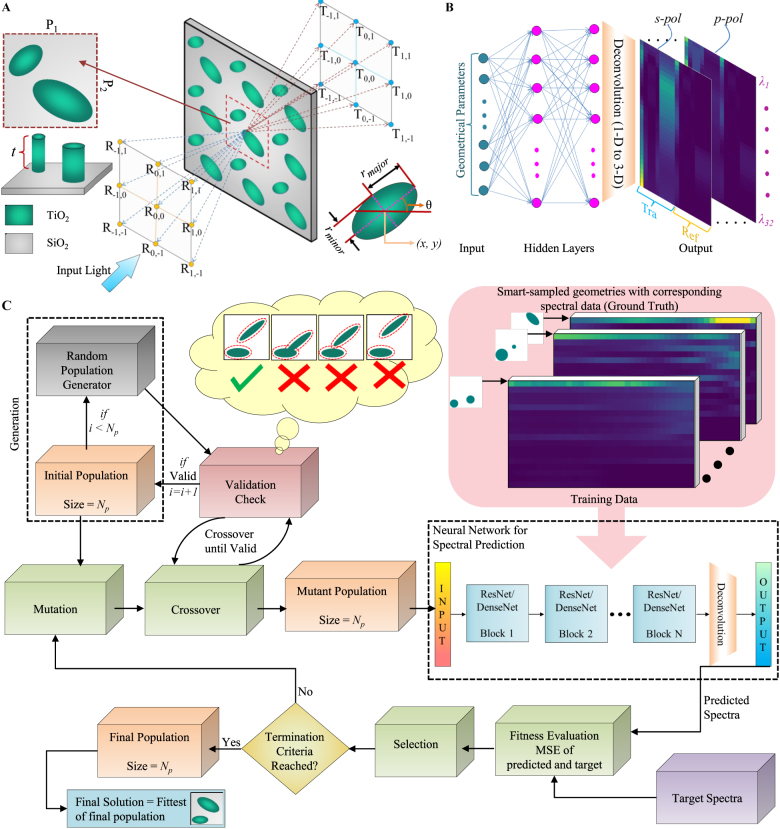
Design of experiments for DL based structure discovery of extended unit cell metagratings. (A) Schematic of the extended unit cell metagrating and its optical response which results in splitting the incoming normally incident beam into various diffraction orders. The structure consists of two elliptical TiO_2_ nano-pillars on SiO_2_ substrate (Insets show the top and perspective views of a single unit cell and the geometrical parameters). (B) A “learned” deep neural network (DNN) model of the structure which predicts the power splitting in various orders over a wavelength range for a given member geometry. (C) Flowchart of learning based surrogate differential evolution (DE) optimization for extended unit-cell metagrating design.

A DE optimization is employed in this work for the inverse design of the metagratings where a randomly initialized set of solutions (called the “population”) are taken and nature inspired techniques like mutation and crossover are performed to produce a new set of solutions [87, 88]. In our previous works [89, 90], where DE was used to optimize nanophotonic structures, the optical response was obtained using a forward solver (*S* ^4^). In this work, we substitute the forward solver with the above discussed DNN based prediction models Surrogate-1 and Surrogate-2 and the associated DE optimization models are referred to as DE_Surrogate-1_ and DE_Surrogate-2_, respectively. Figure 2C shows the block diagram of the surrogate optimization. The DE optimization searches for a design that closely matches a targeted response (specified across orders, wavelengths and input polarization). The fitness (*η*) in the optimization is calculated by taking the mean of squared error between the obtained transmittance (*s*) and target spectra (*τ*) as given below.

(1) $$\eta =\frac{1}{2N_{o}N_{\lambda }}\overset{N_{o}}{\underset{i=1}{\sum }}\overset{2}{\underset{j=1}{\sum }}\overset{N_{\lambda }}{\underset{k=1}{\sum }}{\left[s\left(i,j,k\right)-\tau \left(i,j,k\right)\right]}^{2},$$

where *N*_o_ = 18 and *N*_*λ*_ = 32 are the number of diffraction orders (both reflection and transmissions) and wavelength samples, respectively.

## 3 Results and discussions

The numerical experiments were performed in the Keras DL platform with a Tensorflow backend on a workstation with an Intel™ i9-7920X CPU with an NVIDIA™ GeForce GTX 1080 GPU card with 128 GB memory. The source code for the implementation, datasets and saved models has been made publicly available at [91]. For ground-truth generation and exact fitness estimation of structures for use in evolutionary algorithms, the Stanford Stratified Structure Simulator [92] (*S* ^4^) was used, which uses the Rigorous Coupled Wave Analysis (RCWA) technique along with the S-matrix algorithm to solve Maxwell’s equations in layered periodic structures. In the *S* ^4^ solver, the number of basic functions parameters was set to 50. For the results presented in this section, the prediction error of a trained model (the mean of squared errors (MSE) between the true and predicted output values for members of the test set) is considered. Models with prediction error values less than 5e-4 were found to give a satisfactory performance (see Figure S-5 in the supporting document for more details).

**Figure 3:**
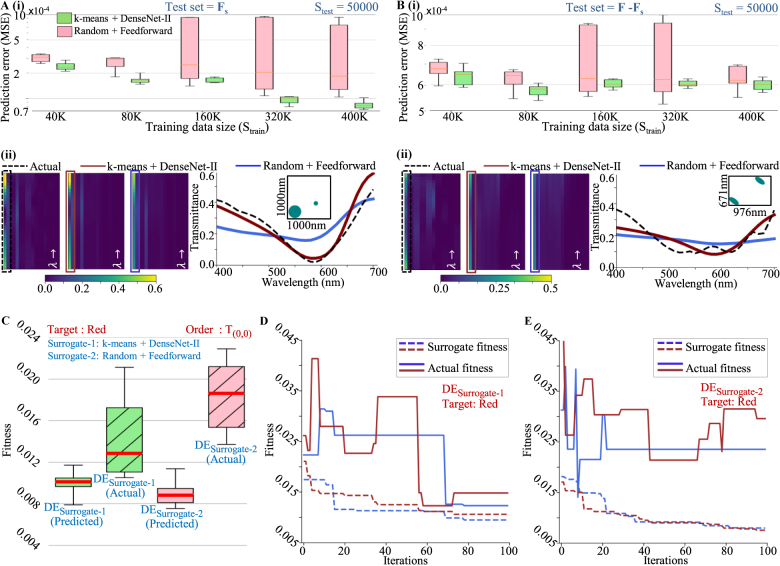
Comparison of the prediction performance on unseen data for two combinations of sampling strategies and network architectures (k-means + DenseNet-I and Random + Feedforward) on two test sets of size 50,000 selected from (A) (**F**_**s**_) and (B) (**F − F**_**s**_), respectively. Statistical comparison of prediction error along with the prediction of one example structure is shown for both cases. (C) Comparison of both trained models’ performances as surrogates in DE optimization. Statistics of multiple runs of DE optimization (100 iterations) for a target design of a polarization-independent color filter (red) are shown. The best surrogate fitness (fitness predicted by surrogates; see Eq. (1)) and the actual fitness values at the end of multiple optimization runs are shown. (D) and (E) Typical progression of fitnesses (with iteration number) for DE_Surrogate-1_ and DE_Surrogate-2_, respectively. For surrogate optimizations, the actual fitnesses (solid lines) and corresponding surrogate fitnesses (dotted lines) are shown.

An extensive comparison of performance was carried out for all combinations of network architectures and sampling strategies discussed above and are documented in Section S-2 of the supporting document. A DenseNet-II network architecture trained with samples selected from **F**_**s**_ with the help of k-means clustering strategy (referred to as “Surrogate-1”) is statistically observed to be having a greater prediction ability as compared to other combinations. Figure 3A and B shows the prediction ability of Surrogate-1 on two unseen testing datasets of size 50 K selected from **F**_**s**_ and (**F − F**_**s**_), respectively. In order to showcase its efficacy, this prediction model is compared with another prediction model “Surrogate-2” which uses a feed-forward network architecture trained with randomly selected samples. The training dataset size is increased from 40 to 400 K for both configurations. All experiments were repeated for 10 times and the distribution of prediction error is plotted in Figure 3A-i and B-i. A clear advantage of prediction quality in Surrogate-1 is observed over Surrogate-2 with mean error, as well as the error variance across multiple runs, are both noticeably lower. Although increasing training dataset size (*S*_train_) beyond 80 K further reduces the prediction error in test set **F**_**s**_, in test set (**F − F**_**s**_) it gives a saturated performance, thus making 80 K a suitable training dataset size. To illustrate, Figure 3A-ii and B-ii show two example structures drawn from both test sets and their actual and predicted optical response. The training dataset size was set to 80 K. Noticeable differences are observed in the prediction of different models with Surrogate-1 outperforming Surrogate-2.

Further, both surrogate models are implemented in designing a surrogate assisted DE optimization. The DE optimization searches for a design that closely matches a targeted response (specified across orders, wavelengths and input polarization). In all the DE optimizations, the population size is fixed to 110 and the initial populations are randomly selected. The DE schemes implemented here are DE/rand/1/bin (mutant is derived through one difference operation between two random solutions and a binary crossover takes place between parent and mutant solution vector to generate the offspring) for exploration. A good convergence rate is observed with mutation rate (*m*_r_) = 0.4 and crossover probability (*c*_p_) = 0.5. All the DE runs are observed to be converging after 100 iterations, making it the termination criterion. The fitness (*η*) in the optimization is calculated as discussed in Eq. (1). Specifically, for illustration, the design of a polarization-independent “Red” color filter is considered under the condition of normal incidence. For this target, Eq. (1) assume the following form:

(2) $$\eta =\frac{1}{2N_{\lambda }}\overset{2}{\underset{j=1}{\sum }}\overset{N_{\lambda }}{\underset{k=1}{\sum }}{\left[s\left(1,j,k\right)-{\text{CMF}}_{\text{red}}\left(k\right)\right]}^{2},$$

where CMF_red_ is the CIE color matching function for red color (sampled at 32 wavelengths).

Figure 3C shows the comparative performance of DE_Surrogate-1_ and DE_Surrogate-2_ for this target. Each DE optimization was repeated 10 times with random initializations and the statistics of the best fitnesses obtained at the end are plotted in Figure 3C. As the surrogate models use DNN predictions of optical responses, the predicted fitness may vary from actual fitness. Therefore, the actual fitness of the best solutions was also calculated for DE_Surrogate-1_ and DE_Surrogate-2_ and plotted. It can be observed that DE_Surrogate-1_ outperforms DE_Surrogate-2_ noticeably for the same training dataset size of 80 K.

The surrogate optimizations perform the search in an approximated fitness landscape. In regions of the parameter space where the prediction capability of the model is poor, there will be a large divergence between actual fitness and predicted fitness. Figure 3E and F shows two typical progressions of an optimization run as indicated by the best obtained fitness value at the end of each iteration for DE_Surrogate-1_ and DE_Surrogate-2_, respectively. The exact fitness of the best individual is also plotted to indicate the divergence between the exact and the approximate fitness landscapes at various junctures of the optimizations. It was observed that the optimizations were converging within 100 iterations. In the case of DE_Surrogate-1_ and DE_Surrogate-2_, the actual fitness is observed to be diverging from predicted fitness; suggesting the degradation in performance. This divergence is caused as the optimization makes the population evolve to solutions where the predicted fitness is low, but the prediction is inferior. As observed in Figure 3E and F, in case of DE_Surrogate-1_, the divergence is less as compared to DE_Surrogate-2_ suggesting a better global prediction ability which results in enhanced optimization capability of DE_Surrogate-1_. Solutions with best fitness in the exact fitness landscape are considered as best fit solutions.

### 3.1 Design of spectral filters and color splitters

In this section, surrogate DE optimization is employed for the inverse design of the following functionalities (for normal incidence); (1) polarization-insensitive RGB color filters; (2) polarization-dependent RGB color filters; and, (3) RGB color splitters. The performance (spectral behavior and color purity) of best-fit solutions obtained with surrogate optimizations (DE_Surrogate-1_ and DE_Surrogate-2_) are compared.

**Figure 4:**
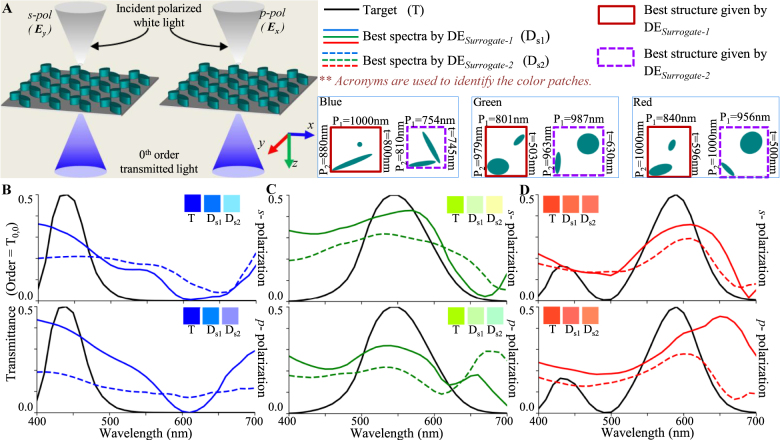
Inverse design of polarization independent transmission mode spectral filters using DE_Surrogate-1_ and DE_Surrogate-2_. (A) Configuration of an example spectral filter. (B), (C) and (D) Transmission spectra of best fit spectral filters obtained by DE_Surrogate-1_ and DE_Surrogate-2_ for designing polarization independent blue, green and red color filters. Inset patches show the colors represented by the spectra in CIE 1931 color space. The unit cell structures obtained with both techniques are shown in color coding for all cases.

For the polarization-insensitive spectral filters (schematic configuration shown in Figure 4A), Eq. (1) assume the following form:

(3) $$\eta =\frac{1}{2N_{\lambda }}\overset{2}{\underset{j=1}{\sum }}\overset{N_{\lambda }}{\underset{k=1}{\sum }}{\left[s\left(1,j,k\right)-\text{CMF}\left(j,k\right)\right]}^{2},$$

where the target CMFs are same for both polarizations; i.e., CMF(1, *k*) = CMF(2, *k*). The results of the inverse design process for all three primary RGB filters are compared across DE_Surrogate-1_ and DE_Surrogate-2_ in Figure 4B–D. It is seen that the designs found by DE_Surrogate-1_ outperform DE_Surrogate-2_ with an improved level of polarization insensitivity and color purity.

**Figure 5:**
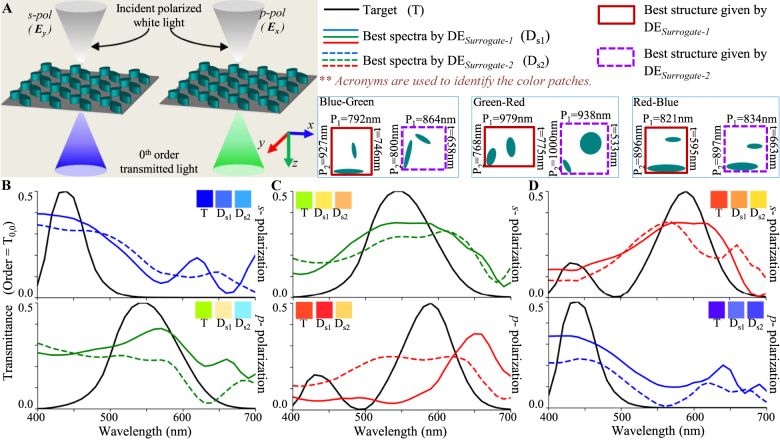
Inverse design of polarization dependent transmission mode spectral filters using DE_Surrogate-1_ and DE_Surrogate-2_. (A) Configuration of an example spectral filter. (B), (C) and (D) Transmission spectra of best fit spectral filters obtained by DE_Surrogate-1_ and DE_Surrogate-2_ for designing polarization dependent (B) blue-green, (C) green-red and (D) red-blue color filters. Inset patches show the colors represented by the spectra in CIE 1931 color space. The unit cell structures obtained with all three techniques are shown in color coding for all cases.

For the polarization-dependent spectral filters (schematic configuration shown in Figure 5A), the fitness is again given by Eq. (3), but the target CMF is different for each polarizations (i.e., CMF(1, *k*) ≠ CMF(2, *k*)). It is seen that the designs found by DE_Surrogate-1_ outperform DE_Surrogate-2_ with higher level of polarization dependence.

**Figure 6:**
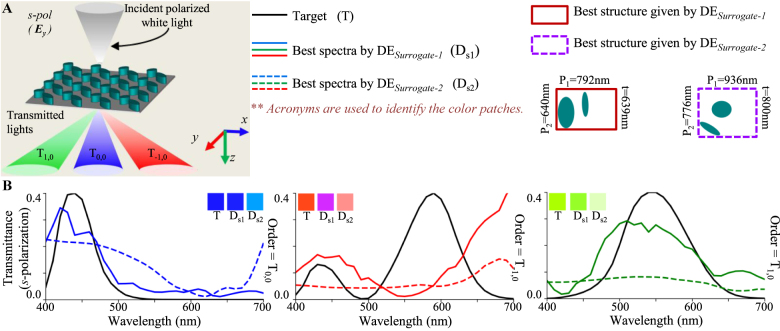
Inverse design of transmission mode color splitters using DE_Surrogate-1_ and DE_Surrogate-2_. (A) Configuration of an example color splitter. (B) Transmission spectra of color splitter designed for s-polarized incidence obtained with DE_Surrogate-1_ and DE_Surrogate-1_. Inset patches show the colors represented by the spectra. The unit cell structures obtained with all three techniques are shown in color coding.

For designing RGB color splitters for s-polarized incident light (schematic configuration shown in Figure 6A), the fitness term in Eq. (1) assume the following form:

(4) $$\eta =\frac{1}{3N_{\lambda }}\overset{3}{\underset{i=1}{\sum }}\overset{N_{\lambda }}{\underset{k=1}{\sum }}{\left[s\left(i,1,k\right)-\text{CMF}\left(i,k\right)\right]}^{2},$$

where the target CMFs are different for each order. For an RGB color splitter where blue, green and red colors are transmitted in *T*_0,0_, *T*_1,0_ and *T*_−1,0_ orders, respectively (Figure 6A), CMF(1, *k*) = CMF_blue_(*k*), CMF(2, *k*) = CMF_green_(*k*) and CMF(3, *k*) = CMF_red_(*k*). The inverse design of this color splitter was carried out using DE_Surrogate-1_ and DE_Surrogate-2_ and the results are compared in Figure 6B. A clear advantage of DE_Surrogate-1_ is observed over DE_Surrogate-2_ in all cases.

Comparing the spectral results of optimized structures in Figures 4, 5 and 6, it can be inferred that DE_Surrogate-1_ is able to follow the target curve better than DE_Surrogate-2_ resulting a better fitness; which indicates a better prediction ability of DE_Surrogate-1_. From the comparison of these colors, it can be observed that in most of the cases, structures optimized with DE_Surrogate-1_ showcase a better color purity as compared to DE_Surrogate-2_; apart from some anomalies in Figure 6B (red). In this exceptional case, although DE_Surrogate-2_ gives structures with better color purity, the transmission peak of structure given by DE_Surrogate-2_ is observed to be considerably small (∼0.15).

The proposed method involves a one-time investment in model creation but significantly reduces the computational time of any optimization objective. The surrogate optimization routine adapted in this work involves a single call (for the best fit solution) to the electromagnetic forward solver in a single iteration; unlike traditional optimizers where the forward solvers evaluate the whole population. The surrogate optimization is found to speed-up the optimization process by “*N*_pop_” times; where “*N*_pop_” is the population size (110 in our case). A detailed account of timing at different junctures of model creation and surrogate optimization are given in Section S-4 of the supporting document. The proposed algorithm is thus useful as the initial investment in model creation can be amortized across several different optimization objectives.

## 4 Conclusions

In conclusion, we demonstrate the advantage offered by densely connected neural network architectures and judicious dataset sampling strategies for learning based nanophotonic structure discovery. In particular, we consider learned models for use in surrogate-assisted evolutionary optimization. The inverse design of optical devices to meet a targeted spectral performance is a general problem and we speculate that the findings of this study can be adopted in inverse design scenarios beyond the example chosen here. Beyond DE considered here, there is a vast array of evolutionary optimization methods used in photonics and it is anticipated that learning based acceleration could be of interest. Although the scope of this work was restricted to parameterized geometry representation and moderate input dimensionality, we speculate on the extension to other problems. Using the methods of dimensionality reduction via the use of autoencoder networks [73, 93], the use of nonparameterized geometry representation can broaden the applicability of this work.

Our previous works [66, 94], have explored different combinations of surrogate models and evolutionary computing by which one can trade off optimality and computational load. In this work, we have considered surrogate-only optimization. When compared with DE_RCWA_, DE_Surrogate-1_ dramatically reduces the computational load but underperforms in terms of optimality. Specifically, choosing surrogate models exclusively for the candidate selection step in evolutionary optimization is recommended in some situations [66, 94].

## Supplementary Material

Supplementary Material Details
