# Supplementary material for: A learning based approach for designing extended unit cell metagratings
Source: Nanophotonics. 2021 Dec 8;11(2):345–58. doi: 10.1515/nanoph-2021-0540 (PMC11501505; doi:10.1515/nanoph-2021-0540)
Supplement: Supplementary file 1 — Supplementary Material Details [file j_nanoph-2021-0540_suppl.pdf]

Research Article

Soumyashree S. Panda and Ravi S. Hegde*

Supporting Document: A learning based approach for designing extended unit cell metasurfaces

https://doi.org/10.1515/sample-YYYY-XXXX
Received Month DD, YYYY; revised Month DD, YYYY; accepted Month DD, YYYY

S-1 Details of deep neural network models' training

S-1.1 Problem encoding

In the first experiment, the relative performance of sampling strategy and network architecture combinations were systematically studied. We took a smaller subset of $\mathbf{F_s}$ by fixing the periodicities and metasurface thickness as given below:

$$\begin{aligned} &P_1, P_2 : 800(nm) \\ &Thickness : 550(nm). \end{aligned} \quad (1)$$

This gave us an 8-dimensional solution space of size 54980 (which was named $\mathbf{F_s^1}$). Models were trained on subsets drawn from $\mathbf{F_s^1}$ and their prediction error (MSE) on the entire set $\mathbf{F_s^1}$ is used to quantify their performance (lower numbers imply better models). As randomness is involved in the dataset sampling as well as during various phases of model training, given combinations of architectures and dataset sampling strategies were assessed over multiple runs.

**Transmittance**									
Index	1	2	3	4	5	6	7	8	9
Diffraction Orders	$T_{0,0}$	$T_{0,-1}$	$T_{-1,0}$	$T_{1,0}$	$T_{0,1}$	$T_{1,1}$	$T_{-1,0}$	$T_{-1,-1}$	$T_{1,-1}$
Reflectance									
Index	10	11	12	13	14	15	16	17	18
Diffraction Orders	$R_{0,0}$	$R_{0,-1}$	$R_{-1,0}$	$R_{1,0}$	$R_{0,1}$	$R_{1,1}$	$R_{-1,0}$	$R_{-1,-1}$	$R_{1,-1}$

Tab. S-1: Index values of different diffraction orders in the 3D encoding.

First, the network hyperparameters (e.g. number of hidden layers, activation function, epochs, initial weights, batch size etc.) were optimized by training a feedforward network and comparing the effect of these parameters on the prediction error. The complete $\mathbf{F_s^1}$ solution space was considered for training and testing ($S_{train} = S_{test} = 54980$). The learning was carried out by minimizing the MSE training loss with a Nadam optimizer. Initial weights are randomly chosen and the batch size is fixed to 256. The number of nodes in the hidden layer was observed to have a negligible impact on learning ability as long it is not very small and was thus fixed to 288 throughout. The number 288 is chosen taking the output dimensionality into consideration ($18 \times 2 \times 32$). 'ReLU' and 'Sigmoid' activation functions are chosen for the hidden and final (deconvolution) layers respectively. It was observed that the hyperparameters that

***Corresponding author: Ravi S. Hegde,** Departmrnt of Electrical Engineering, IIT Gandhinagar, India, 382355 e-mail: hegder@iitgn.ac.in

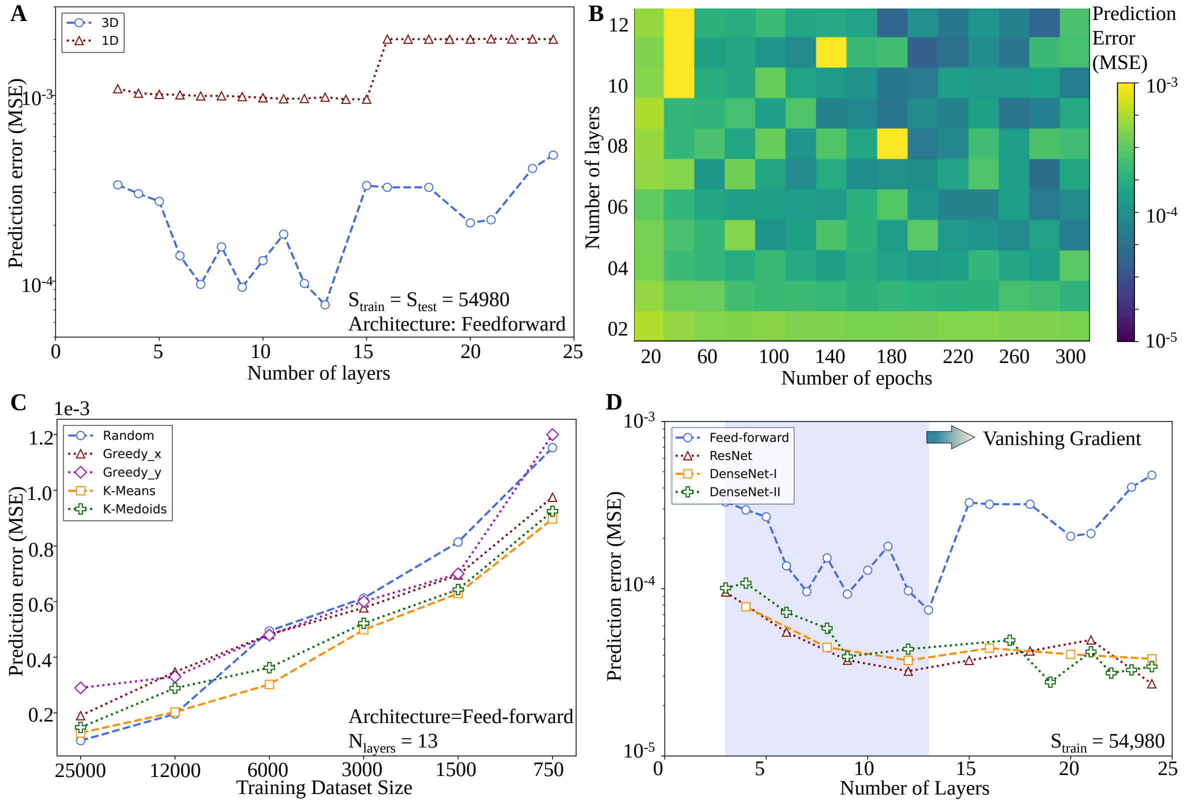

Fig. S-1: **A**: Comparison of testing error for two kinds of encoding: 1D and 3D (discussed in section II of the main article) with increasing the number of hidden layers. **B**: Comparison of testing error (MSE) with respect to different numbers of epochs and hidden layers in a feed forward network architecture. **C**: Performance of 13-layered feedforward network architecture when training dataset size is gradually halved. The training data are sampled from $\mathbf{F_s^1}$ with Greedy_x, Greedy_y, k-means and k-medoids sampling strategies. **D**: Performance of different network architectures with an increasing number of layers. Entire solution space of $\mathbf{F_s^1}$ is considered for training and testing in **A,B,D**.

impact the learning rate greatly are the number of epochs and the number of hidden layers. In a feedforward network, the number of hidden layers is gradually increased from 3 to 25 and all of these networks are trained for 20 to 300 epochs. As discussed in section IIA of the main text, two kinds of output encoding are possible: (1) 1D encoding and (2) 3D encoding. The index values of different diffraction orders in the 3D encoding are given in table S-1.

Figure S-1**A** shows the comparison of validation losses for both the encodings when predicted by simple feedforward networks. The number of hidden layers was increased from 3 to 24. A clear advantage of 3D encoding can be observed over 1D encoding with 1D and 3D encoding performing best for networks with 13 and 15 hidden layers respectively. In 3D encoding, the best prediction (prediction error $\approx$ 1e-4) is observed to be almost 10 times better than the same of 1D encoding (prediction error $\approx$ 1e-3). Figure S-1**B** shows the minimum prediction error obtained by a 13-layered feedforward network. Each epoch-layer combination is repeated 10 times with different initialized weights. From figure S-1**B** it can be observed that in every network the prediction ability almost saturates after 200 epochs; thus the number of epochs is fixed to 200 in all experiments.

S-1.2 Dataset sampling

The default random sampling strategies assign a uniform probability for each member in $\mathbf{F_s^1}$. The next sampling strategy, greedy sampling (GS) [1], works in a sequential manner where the nth sample X_n uses the information from the previously selected samples X_1, $X_2 \cdots X_{n-1}$ as shown in figure S-2**A-iii**. In greedy sampling, from the unlabeled

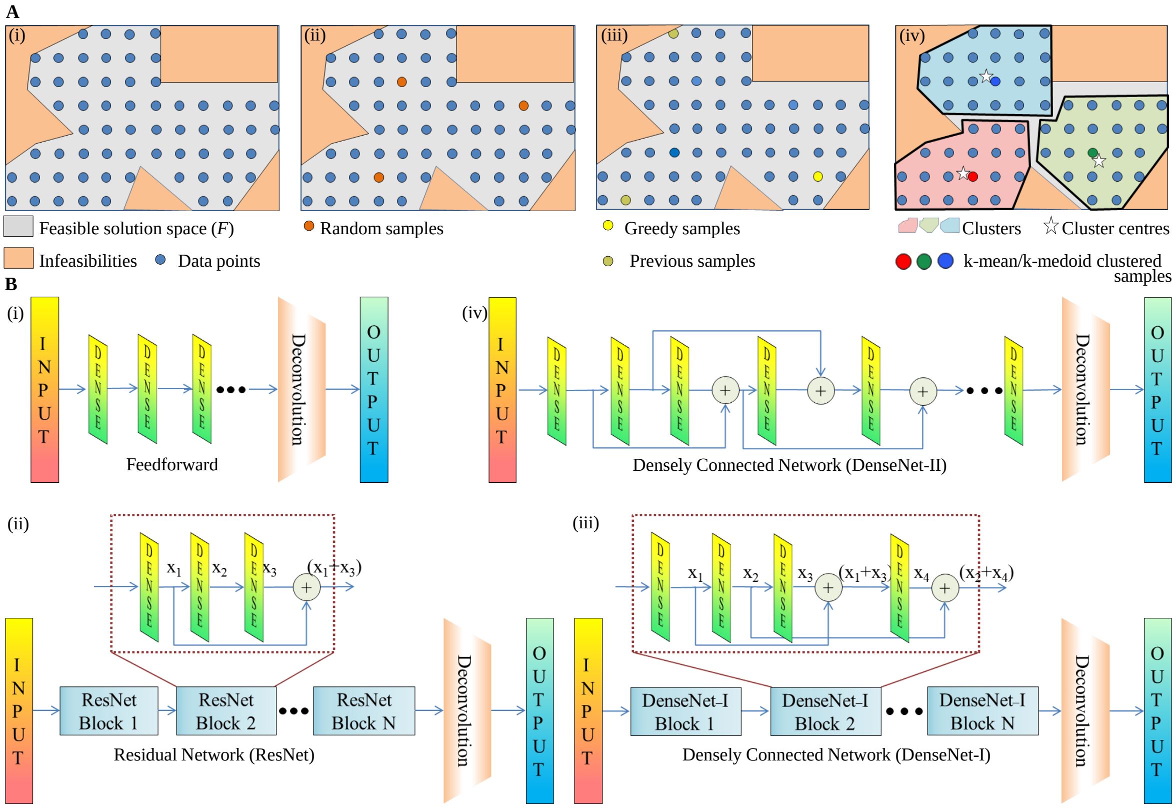

Fig. S-2: A: Various strategies to sample thefeasible solution space. (i): Partitioning of the entire input parameter space (**H**) into "feasible" (**F**) and "infeasible" regions. Uniform sampling of the "feasible" subregions results in a sampled unlabeled set $\mathbf{F_s}$. (ii, iii and iv): Random, greedy and centroid-based sampling procedures to create labeled subsets of $\mathbf{F_s}$ for model training and/or validation. **B**: Four deep neural network (DNN) architectures ((i) feed-forward, (ii) ResNet, (iii) DenseNet-I and (iv) Densenet-II) investigated in this work.

dataset **U**, the nth sample which is far away from the previously labeled (n-1) samples is selected as follows:

$$\arg \max_{\forall X_j \in U} \left(\min_{i<n} \text{dist}(X_i, X_j) \right) \tag{2}$$

This method only fulfills the criterion of *'diversity'*. Another GS strategy Greedy_y was adapted which is based on active learning where the nth sample is selected in a greedy way, but the euclidean distances in the predicted space (Y_{pred}) are compared.

The locations of infeasible points make the solution space irregularly distributed. Therefore, two clustering strategies are implemented; k-means and k-medoids clustering [2] as shown in figure S-2**A-iv**. In contrast to greedy sampling, the clustering methods are nonsequential and the sampling takes place in one step. The quantized feasible solution space is divided into clusters based on the geometric proximity between points; imparting the diversity of the clusters. Each cluster is represented by a cluster centre which serves as a prototype of the cluster. In k-means clustering, the cluster centres are defined by taking the mean of geometrical parameters of all cluster members. However, the cluster centres may fall in the "infeasible" regions. Therefore, another step is included where a solution from $\mathbf{F_s}$ that is nearest to the cluster centre is redesignated as the cluster centre and is subsequently labeled. The k-medoid clustering is similar to k-mean but creates a centroid from the existing cluster members which are known to be in the "feasible" region. Both the clustering methods enhance the *representativeness* and *diversity* of the training data.

The performances of all these sampling strategies are compared in figure S-1**C**. In each case, a network is trained for a fixed size of training data (S_{train}) selected from $\mathbf{F_s^1}$ using the mentioned sampling strategies. Each experiment was repeated 10 times and median prediction errors were compared. The training data sizes are roughly halved until

S_{train} = 750. It can be observed that for smaller dataset sizes (S_{train} < 12000), all the smart sampling strategies perform better than random sampling with k-means clustering performing best for all cases. Both GS strategies, Greedy_x and Greedy_y, are observed to have almost similar performance; therefore only Greedy_x is further explored. In the main text, the term greedy sampling refers to Greedy_x.

S-1.3 Network architectures

Deep neural networks (DNN) with simple feedforward architectures (figure S-2**B-i**) are the most prevalent. They are able to map the input-output relations for a number of problems and their prediction accuracy can be increased to some extent by increasing the number of hidden layers (which increases the number of trainable parameters). For a dataset of fixed size, indiscriminately increasing the number of trainable parameters is known for leading to overtraining. Even when the dataset sizes are adequate to prevent overtraining, simply increasing the number of feedforward layers results in plateauing (and in some cases degradation) in prediction accuracy despite increasing dataset size. This is the problem of "vanishing gradients" (wherein the gradient information (a partial derivative of error functions with respect to weights) becomes vanishingly small) that is well known in the literature. Residual learning (ResNet) [3] addresses this issue of vanishing gradient with identity shortcuts that propagate the gradient by skipping one or more layers (figure S-2**B-ii**). These skip layers are built on the concepts of Pyramidal cells found in the cerebral cortex where the neurons skip connections with the intermediary layer to form longer connections. These skip layers neither contribute to the network hyperparameters nor take any additional computational power when added to the architecture. In DenseNet architectures [4], identity mappings are further exploited by directly connecting all layers; that not only alleviates the vanishing-gradient but also strengthens feature propagation by reusing them. In this work, we have explored two DenseNet architectures. (1) DenseNet-I architecture where the network is built by cascading dense blocks as shown in figure S-2**B-iii**. Each dense block consists of two skip layers. (2) DenseNet-II architecture where the skip layers are repeated throughout as shown in figure S-2**B-iv**.

In all the architectures, the number of hidden layers was gradually increased up to 24 and the prediction errors were compared. The training was carried out 10 times for each case (with different weight initialization) and minimum prediction errors obtained are plotted in figure S-1**D**. In all the cases, ResNet, DenseNet-I and DenseNet-II architectures are clearly observed performing better than feed-forward architectures; with DenseNet-II performing the best when N_{layers} = 19. The prediction error in the feed-forward network diminishes when N_{layers} = 13 and then starts increasing further; suggesting the vanishing gradient. However, ResNet, DenseNet-I and DenseNet-II continue performing better until N_{layers} = 24. Therefore, the number of layers is fixed to 13 for feedforward network architectures and 24 for other architectures in the further part of the work. Unless otherwise mentioned, the dense layers consist of 288 neurons. For comparison, all these network architectures along with feed-forward networks were trained and validated with the complete solution space of $\mathbf{F_s^1}$.

S-2 Performance comparison of different sampling-architecture combinations

Specifically, the experiment had four network architectures and four sampling strategies resulting in sixteen different combinations each for six different training dataset sizes (S_{train}) and figure S-3 shows the outcome of this experiment. For a fixed training dataset size (S_{train}), 10 different datasets are generated with each of the four sampling strategies. Prior to training, all neural network models of a given architecture were initialized to the same set of initial weights. The combination with the lowest median values and smallest box (minimized variance) can be considered as the preferred combination as it gives the highest probability of training a model with the least effort for a given training dataset size. From figure S-3, the first obvious observation is that the errors gradually decrease as the training dataset size increases. A second observation is that in almost all groups, the proposed densely connected ResNet, DenseNet-I and DenseNet-II architectures outperform simple feedforward architecture in almost every case. The outperformance is most pronounced for smaller datasets. The k-mean and k-medoid clustering methods are seen to have a clear

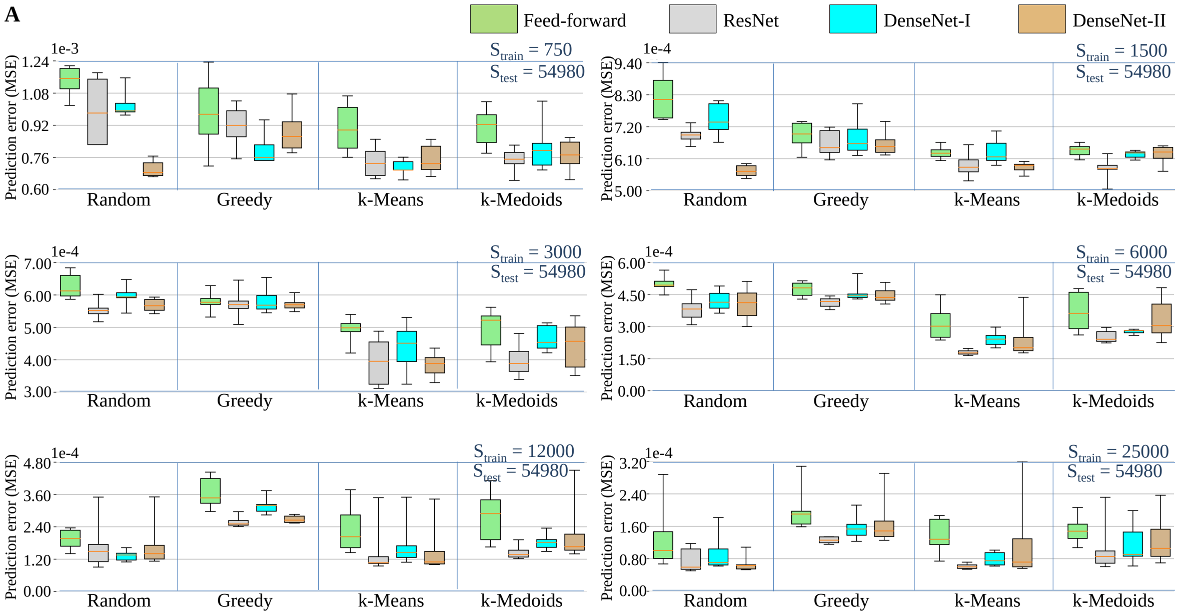

Fig. S-3: Comparison of various *network architecture* + *dataset sampling strategy* combinations grouped according to training dataset size (S_{train}) **A**: Box plots showing the dispersion of prediction errors for a given combination (boxes are color coded with the network architecture to aid the reader). Median prediction errors are represented with horizontal line inside the boxes. The prediction errors are calculated on the full set ($\mathbf{F}_s^1$) (i.e. $S_{\text{test}} = 54980$).

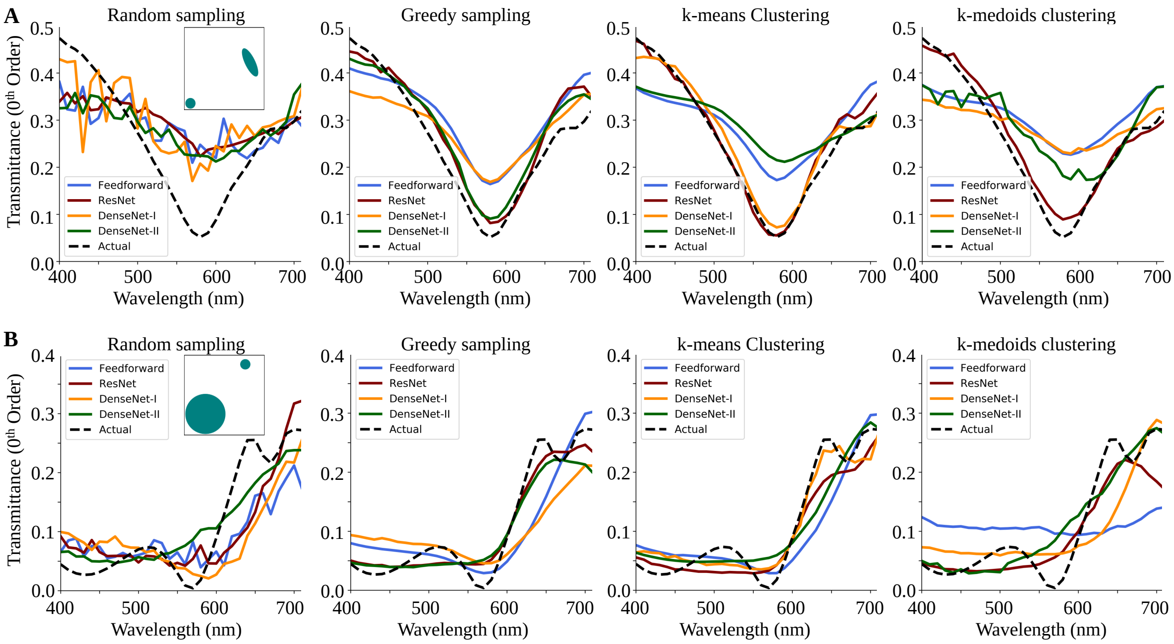

Fig. S-4: Model predictions of the 0th order transmittance of two example structures (shown in insets). The learning is performed by training four network architectures (feedforward, ResNet, DenseNet-I and DenseNet-II) with 6000 labeled datasets selected from ($\mathbf{F}_s^1$) by different sampling strategies (Random, Greedy, k-means and k-medoids).

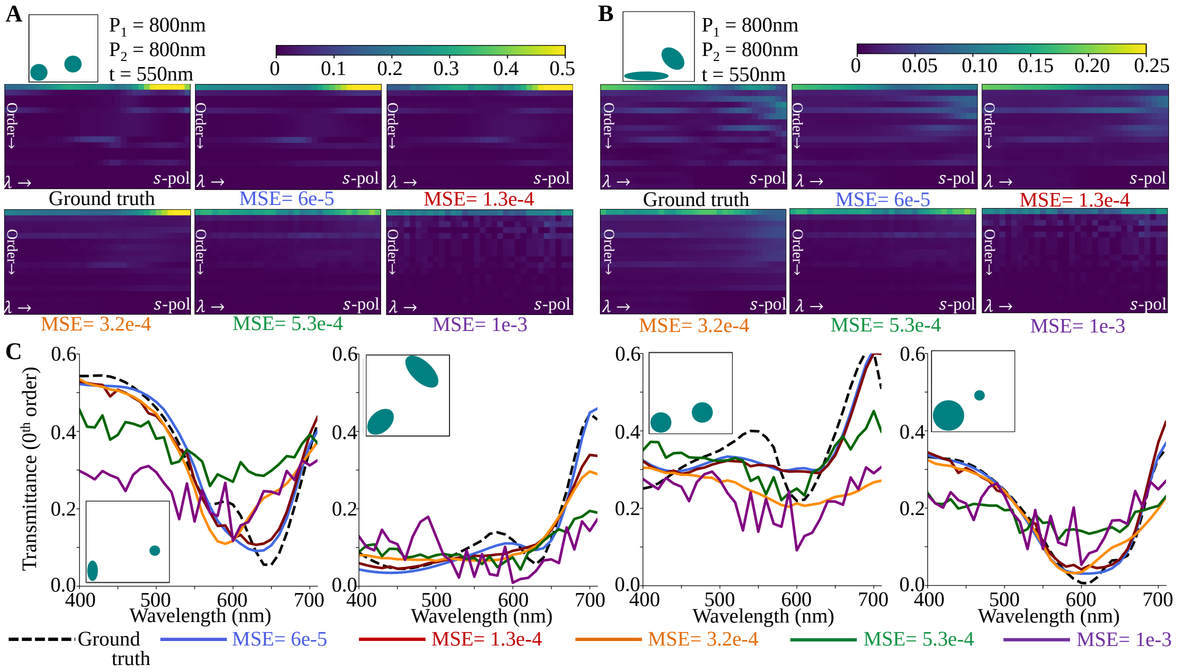

Fig. S-5: Example structures (geometries given in insets) are taken and prediction accuracies of five DNN models (each of different validation loss) are compared with true values. **A**,**B**: Comparison of transmittance/reflectance at different orders for *s*-polarized incidence in two different structures. **C**: Comparison of the predicted and actual 0th order transmittance spectra of four different structures when subjected to *s*-polarized incidence.

advantage in terms of median prediction errors over random or greedy sampling strategies, especially, for smaller training datasets. Finally, the combination of k-mean clustering with DenseNet-II architecture shows minimized variances (box size) for all training data sizes; suggesting more reliable training. To illustrate, figure S-4**A**,**B** show two example structures drawn from $\mathbf{F_s^1}$ (shapes given in insets) and their actual and predicted 0th order transmittance for s-polarized incidence for a training dataset size of 6000 (which is roughly 10% of the full set). Noticeable differences are seen in the prediction of different models with the observation that the combination of random sampling and feedforward architecture appearing to be the worst performer.

S-2.1 Estimating global prediction accuracy of trained models

Any trained neural network model is expected to give a good prediction of optical behavior for all the elements in the set (**F**). However, for any given model, it is impossible to thoroughly quantify its global prediction accuracy considering that **F** is an infinitely large set. Typically the solution space is sampled and the samples are divided into training and testing datasets. The learning process is carried out with the help of training sets and validated on the testing sets and the accuracies on the test sets are considered to be the estimated global prediction accuracy. However, as the training dataset size becomes smaller and smaller, the estimation gets poorer and poorer. In this work, a quantized solution space ($\mathbf{F_s}$) is generated by sampling the geometrical parameters as discussed in S-1.2. The following datasets are considered for estimating a model's prediction ability, especially its' global prediction accuracy.

1. Testset-1: A subset of $\mathbf{F_s}$.
2. Testset-2: A subset of the continuous set ($\mathbf{F} - \mathbf{F_s}$).
3. Testset-3: A subset of the continuous complete feasible solution space (**F**).

While Testset-1 takes samples from the quantized solution space itself, Testset-2 represents interpolated samples followed by checking for feasibility. Evolutionary optimization techniques search the complete solution space for global maxima/minima. Therefore, a trained DNN model used as a surrogate for fitness evaluation in evolutionary

optimization provides an estimate of its global prediction accuracy. Testset-3 is thus a dynamically generated set unlike sets 1 and 2.

S-3 Prediction quality in the perspective of testing error

In order to put the testing error values into the perspective of prediction quality, figure S-5 compares the spectra of some example structures (from $\mathbf{F_s^1}$) predicted by five different DNN models. The prediction error values of the five models are 6e-5, 1.3e-4, 3.2e-4, 5.3e-4 and 1e-3 respectively. Figure S-5**A, B** show two example structures and the heat maps of their transmittance/reflectance in all diffraction orders for s-polarized incidence predicted by all five models. In both cases, the predicted heat-maps are observed to have a good similarity with true values until prediction error = 3.2e-4. Figure S-5**C** shows the 0th order transmittance of some other example structures for s-polarized incidence (geometries given in insets). Here also a good agreement can be observed between the predicted and true values until prediction error = 3.2e-4. For prediction error values 5e-4 and beyond, the prediction accuracy is observed to be degraded (as can be observed from figure S-5). Therefore, as a rule of thumb, prediction error $\leq$ 5e-4 is the necessary condition a model needs to fulfill for being considered adequate.

Bibliography

References

[1] Hwanjo Yu and Sungchul Kim. Passive sampling for regression. In *2010 IEEE International Conference on Data Mining*, pages 1151–1156. IEEE, 2010.

[2] Rui Xu and Don Wunsch. *Clustering*, volume 10. John Wiley & Sons, 2008.

[3] Kaiming He, Xiangyu Zhang, Shaoqing Ren, and Jian Sun. Deep residual learning for image recognition. In *Proceedings of the IEEE conference on computer vision and pattern recognition*, pages 770–778, 2016.

[4] Gao Huang, Zhuang Liu, Laurens Van Der Maaten, and Kilian Q Weinberger. Densely connected convolutional networks. In *Proceedings of the IEEE conference on computer vision and pattern recognition*, pages 4700–4708, 2017.
